# Comparison of the Turn Switch Time Points Measured by Portable Force Platforms and Pressure Insoles

**DOI:** 10.3389/fspor.2020.00002

**Published:** 2020-01-23

**Authors:** Aaron Martínez, Kosuke Nakazato, Peter Scheiber, Cory Snyder, Thomas Stöggl

**Affiliations:** ^1^Department of Sport and Exercise Science, University of Salzburg, Salzburg, Austria; ^2^Faculty of Information Media, Hokkaido Information University, Ebetsu, Japan

**Keywords:** event detection, force binding, GRF, pressure, sensor, ski

## Abstract

Several methods to determine turn switch points during alpine skiing using the vertical GRF exist in the literature. Although comparative studies between pressure insoles (PI) and force platforms (FP) have been conducted, there are no reports comparing the detected time points. Yet, these sensors and methods have been used interchangeably. This study aims to compare the turn switch time points with both sensors and various methods. Twenty skiers performed turns with FP and PI for two different ski styles (high and low dynamic turns). Three different assessment methodologies were compared: minima, functional minima, and crossings. Bland Altman and repeated measures ANOVA were used to assess statistical differences. Main effects of sensor and method were observed (*p* < 0.001). Although there was a low effect size (${\text{η}}_{p}^{2}$ = 0.013) between FP and PI, the 95% CI yielded values representing >30% of the turn duration. A large effect size (η^2^ = 0.153) was found between the crossing method and the minima and functional minima methods. This indicates that those methods assess different events during the turn switch phase. In conclusion, the sensors and assessment methodologies compared in this study are not interchangeable with the possible exception of the minima and functional minima assessed with FP.

## Introduction

In sports, the determination of performance usually depends on small details. Consequently, qualitative assessment of the factors influencing performance is necessary for both recreational and elite levels. The characteristics of alpine skiing make it challenging to study the different features related to performance, injuries, or coaching. Over the last decades there have been several studies using sensors that could collect data while skiing (Müller et al., 1998; Supej et al., 2008; Stricker et al., 2010; Supej, 2010; Nakazato et al., 2011; Hirose et al., 2013; Nemec et al., 2014; Falda-Buscaiot and Hintzy, 2015). To properly assess the specific details influencing alpine skiing such as edge angle, symmetry or turn phases (Müller and Schwameder, 2003; Spörri et al., 2012; Supej et al., 2013; Hebert-Losier et al., 2014), it is necessary to segment the ski runs into the basic units, ski turns. In order to calculate those metrics, it is essential to determine precisely when each turn begins (Spörri et al., 2012).

The typical motion during alpine skiing consists on a cyclic loading and unloading phase for each turn. The up-unweighting phase is characterized by a load shift from the outer to the inner ski (Müller and Schwameder, 2003). This shift is suggested to correspond with the moment of edge change, and consequently the turn switch point (Nakazato et al., 2011). Accordingly, the ground reaction force (GRF) is frequently used to determine turn switches (Nakazato et al., 2011; Spörri et al., 2015; Yu et al., 2016). Two sensors have been used indiscriminately to measure GRF: portable force platforms (FP), and pressure insoles (PI). Although FP are the gold standard for force measurement, PI presents some advantages facilitating the data collection process. PI are generally easy to use, wireless, almost non-obtrusive, while FP require extra mechanics, wiring and energy supply. Differences have been found between the force values measured with both sensors during skiing (Nakazato et al., 2011). The PI tend to underestimate the force values from 21 to 54% depending on the phase of the turn, the skier's level, the slope, and the skiing style (Nakazato et al., 2011). Additionally, the force application point and the center of pressure have been shown to follow different patterns, accentuated in the mediolateral direction (Nakazato et al., 2013). These differences are likely due to the sensor locations, as plantar pressure systems do not measure a significant component of the GRF that is transferred through the ski boot cuff (Stricker et al., 2010; Nakazato et al., 2011). Although comparisons between FP and PI have been done regarding force magnitude and application point, to the authors knowledge there are no studies comparing the time point of the turn switch based on GRF measures.

Apart from the various sensors, several methods to assess the turn switch point based on GRF have been used. Nakazato et al. (2011) used the minimum value of the vertical GRF, representing the point with the minimum load. Although data was collected from both FP and PI, the determination of the turn switch point was only based on the total force combined from both legs assessed with FP. Spörri et al. (2015) detected the beginning and end of each turn based on what they called the “functional minima” of the GRF during the turn switch. To calculate the functional minima, they selected the force values below a certain threshold for each run. The threshold was set as the highest minima among all the summed left and right turn force curves. They defined the turn switch point as the midpoint between the first and the last time points of each turn below this threshold. The advantage of this assessment methodology is that it avoids possible misdetections of turn switch points due to noise or vibrations. Finally, between turns there is a load transmission from the outside to the inside leg. During this phase, there is a point where the load is equal for both sides. It has been suggested that this point could correspond with the turn switch point (Müller and Schwameder, 2003; Yu et al., 2016).

In order to determine if the FP and PI measurement systems can be used interchangeably for turn detection in alpine skiing, this study aims to compare the time points of turn detection between FP and PI using the different methodologies previously proposed. A second aim is to compare those turn detection methodologies and evaluate if they detect the same events during alpine skiing.

## Materials and Methods

To assess the agreement of the turn switch point detection between various assessment methodologies measured with FP and PI, a study was designed where skiers performed alpine skiing turns in two styles (high and low dynamic turns).

### Participants

A total of 20 skiers (18 males and 2 females; Mean ± SD: Age = 24.7 ± 3.5 years; Height = 1.78 ± 0.08 m; Weight = 73.7 ± 9.2 kg) took part in the study. Before the measurement, all participants were informed in detail about the testing procedures, as well as possible benefits and risks of the investigation prior to signing the consent form approved by the local Ethics Committee. The experiment was conducted in accordance with the Declaration of Helsinki.

The measurements were performed for 10 days within 2 weeks. The snow temperature was consistent across all measurement days with a mean temperature of −1.1 ± 2.2°C. The mean air temperature was −0.4 ± 4.8°C. The upper part of the measurement slope had an inclination of 23° while the bottom part had an inclination of 15°. The slope was machine groomed daily resulting in a compact layer of natural and artificial snow.

Two different skiing styles were performed. The skiing technique “Carving in Short Radii” (Wörndle et al., 2011) (which is characterized by a short radius turn and dynamic vertical movement) was defined as high dynamic turns. The technique “Parallel Ski Steering in Long Radii” (Wörndle et al., 2011) (which is characterized by a long radius skidded turn and less dynamic movement) was defined as low dynamic turns. Two different courses were set. While the vertical distance between gates was kept constant for both techniques (between 14 and 17 m depending on the inclination), the corridors were 5 and 10 m for the high dynamic and the low dynamic turns, respectively. Short poles were set into the slope as gates. After a warm up run, each skier performed one run per skiing style in a randomized order. Each run consisted in 20 double turns.

### Equipment

**Figure 1:**
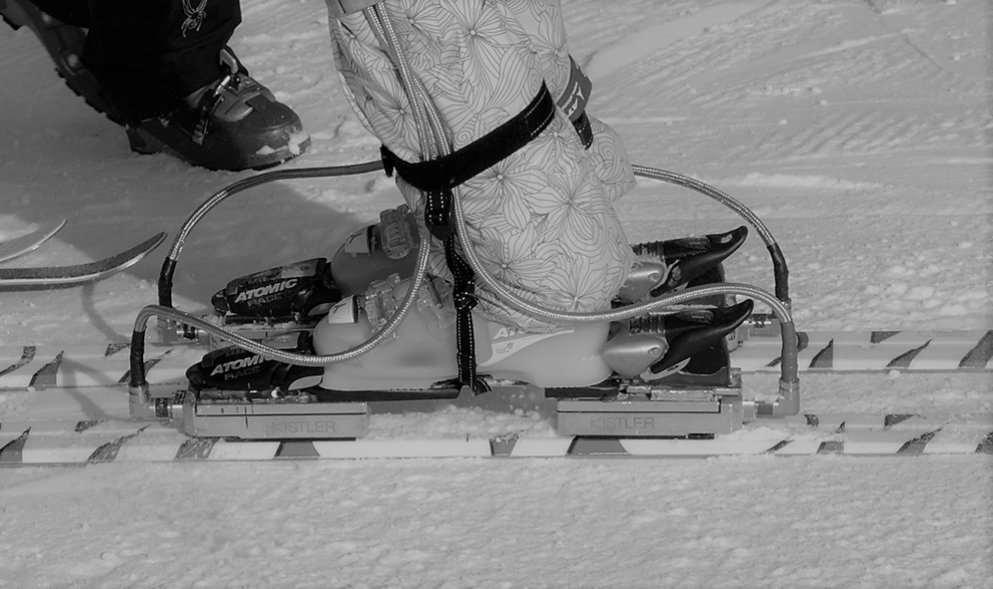
Set up of the FP between the bindings and the skis.

Two different skis were utilized. To properly select the skis, the participants level were assessed based on the Austrian ski teaching concept (Wörndle et al., 2011) by an accredited instructor. The skiers classified as intermediate (*n* = 10) used recreational skis (length = 169 cm, radius = 15 m) and the skiers classified as experts (*n* = 10) used racing skis (length = 184 cm, radius = 23 m). Regardless of the assessed level, all skiers were able to properly perform the required ski styles. The skiers used their own ski boots. The skis were equipped with a portable FP system and a pair of PI were placed inside the liners (Figure 1).

#### Sensors

The FP system consisted of four dynamometers (Kistler, Winterthur, Switzerland) mounted under the toe and heel binding of each ski. Each dynamometer weighed 0.9 kg, was 36 mm high, and consisted of a top and bottom plate connected by three three-dimensional piezoelectric force transducers. Amplifiers, power supply, supply box, and data loggers (4 kg) were carried in a backpack worn by the skiers (see detailed description; Stricker et al., 2010). The PI (Pedar, Novel, Munich, Germany) were formed by 99 cells, each of them including one capacitive sensor. The PI were located inside the liners of the ski boots and the proper size was selected for each skier and replaced their normal insoles. All pressure insoles were calibrated prior to the measurements following the manufacturer's instructions. The Novel data logger, battery pack, and trigger switch were carried in a belt (1 kg in total) attached to the Kistler backpack.

Both systems recorded at 100 Hz, which represents the maximum sampling rate of the PI. In order to synchronize the two systems, participants were asked to stomp at the beginning and end of every run.

### Data Analysis

In the current study, three turn detection methodologies were applied to the data collected simultaneously with both measurement systems (FP and PI). Prior to the application of the turn detection methodologies, the data sets where synchronized (based on GRF peaks produced by the stomps). The force values of the PI are the sum of the force from each cell, which is obtained multiplying its pressure by its area.

**Figure 2:**
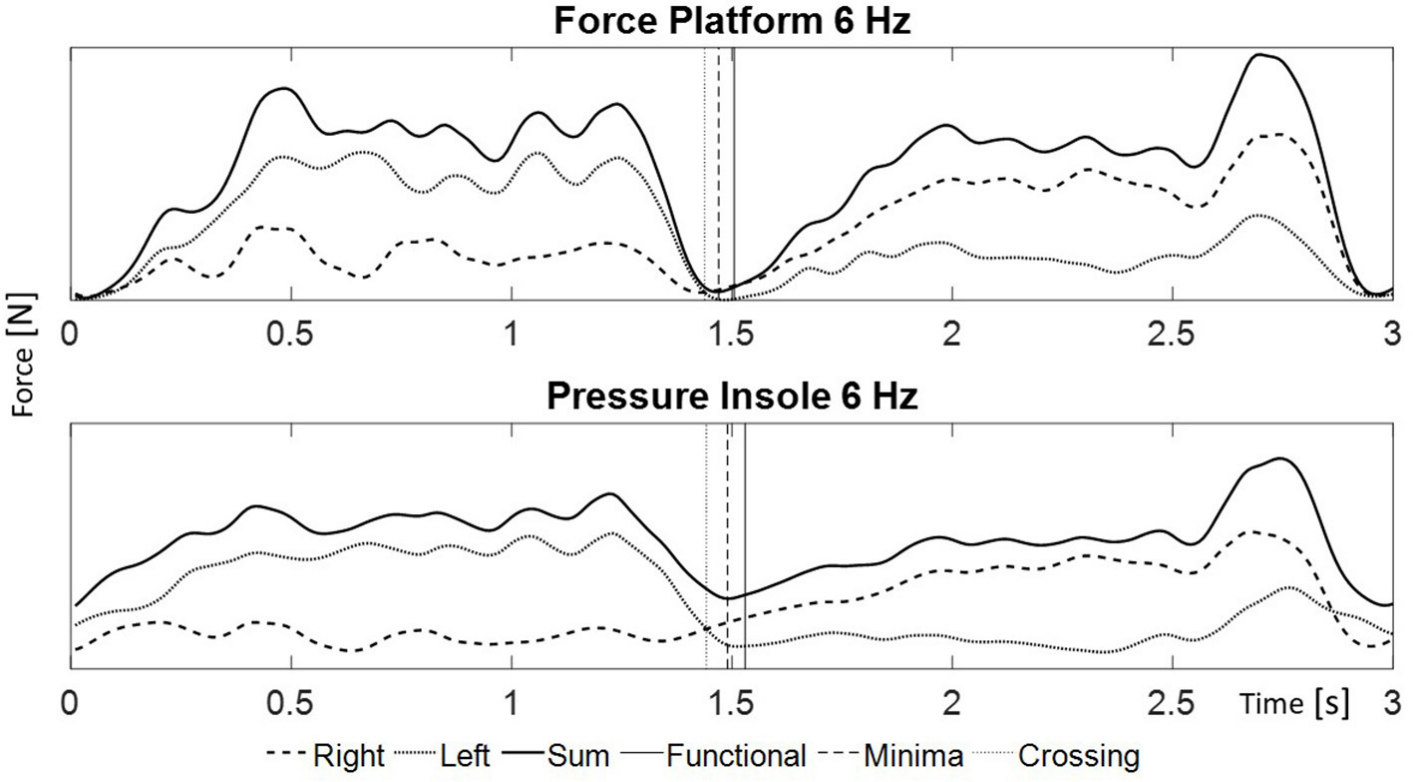
Example of the signals during a turn: the right foot (dashed line), the left foot (dotted line), and the sum (solid line). In the upper graph GRF measured with the FP placed under the bindings. In the lower graph force calculated from the pressure values from the PI between the boot and the foot. The vertical lines represent the turn switch points assessed with the different methods: minima (dashed line), functional minima (solid line) and crossing (dotted line).

The three different turn switch methods assessed were (Figure 2):

1. *GRF minima*. This method uses the minimum value of the vertical component of the GRF determined by the sum of forces from both legs (Nakazato et al., 2011).
2. *GRF functional minima*. This method approximates the point of the minimum value of the GRF summed from both legs based on the cyclic loading and unloading pattern. It avoids possible misdetections of the turn switch point due to noise or vibrations (Spörri et al., 2015, 2016; Kröll et al., 2016).
3. *GRF crossing*. The turn switch point is determined by the point when the magnitudes of GRF of both legs are equal (Müller and Schwameder, 2003; Yu et al., 2016).

In addition, 1, 3, and 6 Hz low pass Butterworth filter frequencies were used and compared. The 3 and 6 Hz filters have been used in previous literature (Spörri et al., 2015, 2016; Martínez et al., 2019), therefore they were selected. The 1 Hz cut-off yielded the lowest bias and range (see section Statistical Analysis) in a pilot study.

### Statistical Analysis

Data processing was performed using IkeMaster (Ike Software Solutions, Salzburg, Austria) and Matlab (Version R2018b, The MathWorks Inc., Natick, MA, USA). To assess the interchangeability and agreement between methodologies and sensors, the bias, and limits of agreement were calculated as proposed by Bland and Altman (1986) (Excel 2016, Microsoft, Redmond, WA, USA). The range was set as the difference between the upper and lower limits of agreement, representing the 95% confidence interval (CI). The comparisons between methods and sensors were performed for the three different cut-off filtering options and for the different intensities: high dynamic turns (*N* = 1,316), low dynamic turns (*N* = 1,156), and for all turns pooled together (*N* = 2,472).

**Figure 3:**
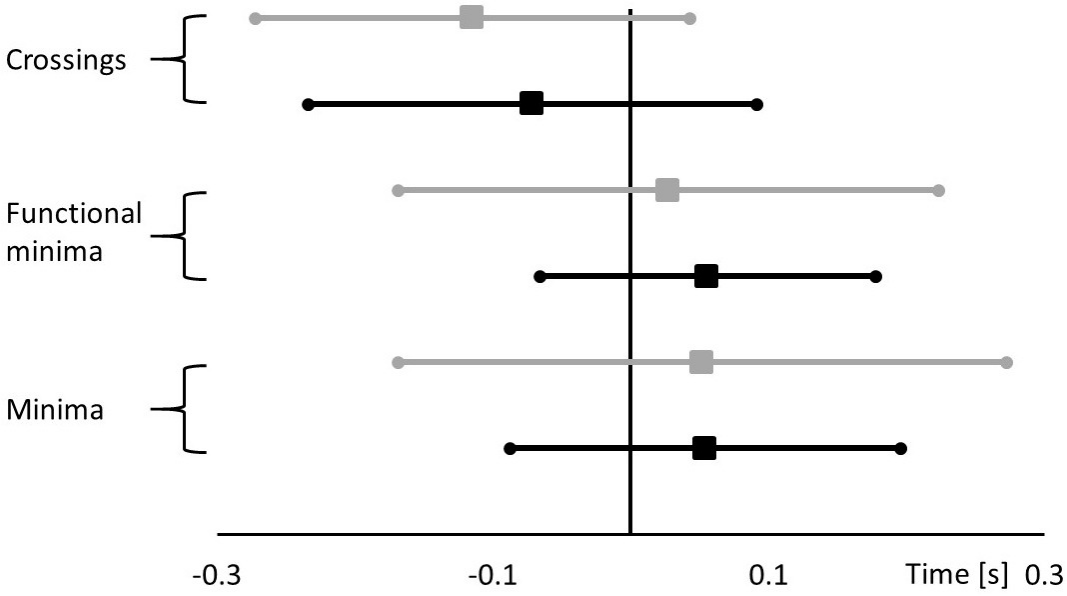
Example of the different moments in time detected by FP (in black) and PI (in gray) represented as the mean ± SD for the 3 different assessing methodologies at 6 Hz with all the turns pooled together.

A repeated measures analysis of variance (RMANOVA) (SPSS Inc., Version 25.0, Chicago, IL, USA) was used to assess statistical differences with respect to system (FP vs. PI), calculation method (minima vs. functional minima vs. crossing), filter (1 vs. 3 vs. 6 Hz), and ski dynamics (high vs. low dynamic turns). Mauchly's Test of Sphericity indicated that the assumption of sphericity was not met, consequently Greenhouse-Geisser correction was utilized. If significant differences were found, Bonferroni's *post-hoc* test was applied. In order to perform the RMANOVA, a reference value was necessary to compare relative turn switch points (i.e., measured turn switch—reference turn switch). This reference was calculated independently for each turn and cut-off frequency as the mean of the 6 measured turn switch points (two sensors by three methods; Figure 3). Eta squared (η^2^) and partial eta squared (${\text{η}}_{p}^{2}$) were used to calculate effect size. Effect sizes were classified as small 0.01–0.06; medium 0.06–0.14, and large >0.14 (Pallant and Manual, 2010). A significance value of α = 0.05 was chosen.

## Results

The agreement between sensors and methodologies are presented in Table 1. Bias and range were consistently lower for the high dynamic turns than for the low dynamic turns in all the conditions assessed, across all systems and methods.

**Table 1:** Bias and range of the 95% CI of the different comparisons between the different assessment methodologies.

			**All**			**Hi**			**Lo**		
			**1 Hz**	**3 Hz**	**6 Hz**	**1 Hz**	**3 Hz**	**6 Hz**	**1 Hz**	**3 Hz**	**6 Hz**
FP vs. PI comparison	Min	Bias	0.009	0.002	0.003	0.032	0.017	0.017	0.056	0.022	0.025
		Range	1.357	1.186	1.229	0.565	0.454	0.526	1.875	1.662	1.704
	Functional	Bias	0.022	0.025	0.029	0.036	0.025	0.023	0.089	0.082	0.087
		Range	1.139	1.090	1.111	0.532	0.502	0.512	1.525	1.470	1.498
	Crossing	Bias	0.015	0.026	0.044	0.010	0.016	0.037	0.020	0.037	0.052
		Range	0.579	0.672	0.789	0.452	0.577	0.684	0.695	0.764	0.893
FP method comparison	Min vs. Functional	Bias	0.002	0.001	0.001	0.002	0.005	0.006	0.001	0.005	0.003
		Range	0.198	0.315	0.391	0.031	0.102	0.211	0.287	0.447	0.525
	Min vs. Crossing	Bias	0.135	0.138	0.126	0.085	0.097	0.076	0.192	0.184	0.182
		Range	0.723	0.736	0.820	0.608	0.660	0.702	0.776	0.775	0.886
	Functional vs. Crossing	Bias	0.137	0.139	0.127	0.087	0.103	0.082	0.193	0.180	0.179
		Range	0.668	0.700	0.778	0.604	0.639	0.673	0.669	0.731	0.837
PI method comparison	Min vs. Functional	Bias	0.012	0.023	0.024	0.007	0.013	0.012	0.032	0.064	0.066
		Range	0.857	0.914	0.933	0.487	0.511	0.550	1.134	1.201	1.212
	Min vs. Crossing	Bias	0.141	0.162	0.167	0.127	0.130	0.130	0.156	0.199	0.209
		Range	1.323	1.237	1.286	0.725	0.696	0.738	1.772	1.638	1.694
	Functional vs. Crossing	Bias	0.129	0.139	0.142	0.133	0.143	0.141	0.124	0.134	0.143
		Range	1.095	1.084	1.112	0.707	0.699	0.701	1.413	1.398	1.444

*Values are expressed in seconds. Three different cut-off frequencies are reported: 1, 3, and 6 Hz. Results are classified by: all turns together, high dynamic turns and low dynamic turns. All, all turns pooled together; Hi, high dynamic turns; Lo, low dynamic turns; Min, assessment method previously referred as GRF minima; Functional, assessment method previously referred as GRF functional minima; Crossing, assessment method previously referred as GRF crossing*.

The comparison between FP and PI for all turns pooled together yielded bias lower than 0.05 s and ranges between 1.09 and 1.36 s, except for the “crossing” method with ranges starting at 0.58 s. The “minima” and “functional minima” methods had the highest agreement for both sensor types (FP and PI). Although the comparison between “minima” and “functional minima” methods had the highest agreement for both sensors, the results were not similar. Including all cut-off frequencies, the bias and range were <0.002 and 0.400 s for the FP, but >0.012 and 0.850 s for the PI, respectively. This behavior was consistent, the comparison between assessment methodologies showed systematically lower bias and range values when the FP system was used.

Results for the mean and standard deviation for each assessment option are shown in Table 2. No main effects of frequency or intensity were observed. Main effects of sensor were observed between FP and PI (*p* < 0.001), but the effect size was small (${\text{η}}_{p}^{2}$ = 0.013). Main effects of method were also observed (*p* < 0.001) with a large effect size (${\text{η}}_{p}^{2}$ = 0.427). *Post-hoc* analysis indicated that all three methods were significantly different than one another. However, while the means of minima and functional minima were statistically different, they were within 0.02 s with a small effect size (η^2^ = 0.001). On the other hand, the differences between both the minima and the functional minima and the crossing method were >0.13 s with a large effect size (η^2^ = 0.153).

**Table 2:** Mean and SD of the different comparisons between the different assessment methodologies.

			**All**			**Hi**			**Lo**		
			**1 Hz**	**3 Hz**	**6 Hz**	**1 Hz**	**3 Hz**	**6 Hz**	**1 Hz**	**3 Hz**	**6 Hz**
FP	Min	Mean	−0.054	0.055	0.052	−0.023	0.026	0.018	−0.089	0.087	0.092
		SD	0.141	0.131	0.138	0.075	0.067	0.065	0.185	0.173	0.182
	Functional	Mean	−0.055	0.055	0.054	−0.029	0.032	0.020	−0.086	0.082	0.092
		SD	0.122	0.116	0.126	0.064	0.060	0.063	0.159	0.152	0.163
	Crossing	Mean	0.072	−0.083	−0.083	0.053	−0.071	−0.067	0.093	−0.098	−0.101
		SD	0.163	0.148	0.148	0.136	0.126	0.120	0.185	0.167	0.170
PI	Min	Mean	−0.051	0.053	0.043	−0.040	0.043	0.050	−0.064	0.064	0.035
		SD	0.221	0.215	0.242	0.109	0.098	0.114	0.301	0.296	0.332
	Functional	Mean	−0.027	0.030	0.031	−0.052	0.057	0.057	0.001	0.000	0.003
		SD	0.196	0.194	0.191	0.103	0.103	0.107	0.262	0.258	0.253
	Crossing	Mean	0.115	−0.109	−0.098	0.090	−0.087	−0.077	0.145	−0.135	−0.121
		SD	0.158	0.145	0.140	0.117	0.111	0.100	0.189	0.173	0.172

*Values are expressed in seconds. Results are classified by: all turns together, high dynamic turns and low dynamic turns. All, all turns pooled together; Hi, high dynamic turns; Lo, low dynamic turns; Min, assessment method previously referred as GRF minima; Functional, assessment method previously referred as GRF functional minima; Crossing, assessment method previously referred as GRF crossing; SD, standard deviation*.

The average turn duration for high and low dynamic turns was 1.66 ± 0.27 and 2.55 ± 0.32 s, respectively. The range between FP and PI for the most similar methodologies (minima and functional minima) represents ~30 and 40% of turn duration for high and low dynamic turns, respectively. The range between minima and functional minima assessed using the PI represents a 33 and 48% of the turn duration for high and low dynamic turns, respectively, and 13% of the turn duration when assessed with the FP, independent of the turn dynamics.

## Discussion

The current study focused on the comparison between time points of the turn switch during alpine skiing assessed with different methodologies and using FP and PI. The results showed that all the methods yielded better agreement when the data was collected using the FP than when it was collected using the PI (Table 1). This trend was accentuated for highly dynamic turns. The higher consistency found with the FP might be due to the improved transmission of the forces from the legs via the boots to the skis, which might not detected by the PI (Supej et al., 2008). The FPs measure the total forces acting between the skis and the bindings, including the force transferred by the boot cuff. On the other hand, the PIs only measures the transmission of forces from the foot to the boot, which will be only part of the force transmitted to the binding. Due to the movement of the skier and the shift in load distribution during the turn, the fraction of the forces measured by PI compared to FP is not constant during the turn (Nakazato et al., 2011, 2013), and could affect the time points where events are detected.

Main effects of sensor were observed between FP and PI, however the effect size was small (0.013). A possible interpretation of those results is that the large number of turns included in the analysis (*n* = 2,472) lead to a type 1 error, were negligible differences are deemed statistically significant. Regardless of the risk of type 1 error, and to the potential misinterpretation of the data, the magnitude of the ranges yielded by the Bland and Altman comparisons show a CI that represents between 30 and 40% of the total turn duration. Consequently, due to the lack of agreement between sensors, these sensors should not be interchanged when comparing turn switch points or metrics measured turn by turn as the cutting point will affect the calculations (Spörri et al., 2012).

The same discussion is relevant for the comparison between the methods. Although there is a main effect of methodology, with a large effect size (0.427), the differences are mostly with respect to the crossings method (>0.13 s; η^2^ > 0.153) and not between the minima and functional minima (<0.02 s; η^2^ = 0.001). Nevertheless, the ranges between minima and functional minima assessed with PI represents >30% of the turn duration which leads to the conclusion that they are not interchangeable. On the other hand, when using the FP, the range represents a 13% of the turn duration, which depending on the aim of the turn detection, could allow for the use of both the minima and functional minima methods interchangeably from FP measurements.

The results indicate that the crossings methodology assesses different events or points in time than the other two methods. Furthermore, the bias and range for this method are considerably higher than the bias and range of the minima to functional minima comparison. A possible explanation for this is that the minima and functional minima methods represent two approaches to the same concept, where the turn switch point corresponds with the point of minimum load from both legs (Müller et al., 1998; Spörri et al., 2015). Consequently, these methods, though different, are more similar to each other than the crossing method. For this method, the point when the load is equal between both legs is used (Müller and Schwameder, 2003). Although both events need to happen during the turn switch phase, they do not necessarily represent the same point in time within the turn switch phase.

Regardless of the apparent similarities in performance between sensors and methods, the results suggest that they are not entirely interchangeable. Although bias' are generally small and suggest that both sensors are assessing the same event, the high ranges (>30% of the turn) highlight the differences of the points actually detected. Unfortunately, we cannot determine which sensor or method would be most representative of the true turn switch point due to the lack of valid reference data, both in the literature and this study. Based on the results, the methodology based on the minima seems to be the most appropriate to apply in the field. It presents the lowest bias values between the two sensors and also the smallest range for highly dynamic turns. On the other hand, both sensors present some advantages and disadvantages. FP showed consistently better results, yet the use of FP in training is not a feasible option. It changes the height of the bindings, adds weight to the skis and implies more weight and wires in the skier's body. For this reason, the implementation of a less cumbersome pressure sensor outside of the boot could potentially solve the problem of the missing forces through the boot cuff and provide reliable data similar to the FP. A future study using a gold standard methodology for the location of the turn switch point needs to be defined and consequently compared with the various methodologies applied for detecting the turn switch.

## Conclusions

The aim of the study was to assess if sensors (FP and PI) or assessment methods (minima, functional minima, and crossings) could be used interchangeably, and according to the results of this study, they are not. The only exception being the minima and functional minima methods assessed with FP. The results of the study also suggest that the use of the FP sensor to determine turn switches based on GRF is recommended. Further research is needed to evaluate the precision of the different systems and determine which assessment method is most correlated with the real turn switch point.

## Data Availability Statement

The datasets generated for this study are available on request to the corresponding author.

## Ethics Statement

The studies involving human participants were reviewed and approved by Ethics Committee of the University of Salzburg. The patients/participants provided their written informed consent to participate in this study.

## Author Contributions

AM and TS: conceptualization and formal analysis. AM, KN, PS, and TS: methodology. AM: software and visualization. AM, CS, and TS: investigation. TS: resources, supervision, and funding acquisition. PS: data curation. AM: writing—original draft preparation. TS, CS, PS, and KN: writing—review and editing.

### Conflict of Interest

The authors declare that the research was conducted in the absence of any commercial or financial relationships that could be construed as a potential conflict of interest.
